# Clinical profiles of older adults in French Caribbean nursing homes: a descriptive cross-sectional study

**DOI:** 10.3389/fmed.2024.1428443

**Published:** 2024-09-17

**Authors:** Denis Boucaud-Maitre, Nadine Simo, Roxane Villeneuve, Christine Rambhojan, Nathalie Thibault, Sarah-Priscilla Joseph, Michel Bonnet, Moustapha Dramé, Larissa Vainqueur, Leila Rinaldo, Laurys Letchimy, Jean-François Dartigues, Matteo Cesari, Yves Rolland, Bruno Vellas, Hélène Amieva, Maturin Tabué-Teguo

**Affiliations:** ^1^Centre Hospitalier Le Vinatier, Bron, France; ^2^University of the French West Indies, EpiCliV Research Team, Fort-de-France, France; ^3^Centre Hospitalo-Universitaire de Martinique, Fort-de-France, France; ^4^Centre Hospitalo-Universitaire de Guadeloupe, Pointe-à-Pitre, France; ^5^Inserm U1219 Bordeaux Population Health Center, University of Bordeaux, Bordeaux, France; ^6^Department of Clinical Sciences and Community Health, University of Milan, Milan, Italy; ^7^Gérontopôle, CHU de Toulouse, Toulouse, France

**Keywords:** nursing homes, older adults, dependency, Caribbean, cross-sectional study

## Abstract

**Background:**

Nursing homes in the Caribbean are scarce and the characteristics of their residents have not been previously documented. This study aimed to describe the clinical profiles of residents living in nursing homes in Guadeloupe and Martinique (French West Indies).

**Methods:**

This is a cross-sectional study of the baseline screening data from the KASEHPAD (Karukera Study of Ageing in nursing homes) study. Clinical characteristics and geriatric scale scores, including the Activities of Daily Living (ADL), Mini-Mental State Examination (MMSE), Mini Nutritional Assessment Short-Form (MNA-SF) and Short Physical Performance Battery (SPPB) were collected and analysed.

**Results:**

A total of 332 older adults were recruited between September 2020 and November 2022. The mean age of the residents was 81.3 ± 10.1, with a male–female ratio of 1:1. Diabetes was reported in 28.3% of the residents, hypertension in 66.6% and heart disease in 18.4%. Dementia was diagnosed in 52.3% of the residents and 74.9% had a MMSE score ≤18. The prevalence of Parkinson’s disease was 9.0%. Additionally, 18.4% were unable to perform any basic activities of daily living (ADL score of 0). The prevalence of physical impairment (SPPB < 8) was 90.0%. One-quarter of the residents were classified as undernourished (MNA-SF score ≤ 7).

**Conclusion:**

Residents in Caribbean nursing homes are younger than in metropolitan France, whereas they present quite similar clinical profiles. Notably, a high prevalence of diabetes, cardiovascular diseases and neurodegenerative diseases was observed. This study represents a preliminary effort to address the knowledge gap regarding the aging trajectories of older adults in the Caribbean and could guide the development of future nursing homes in these countries.

## Introduction

Demographic trends indicate a steady decline in both birth and mortality rates across Caribbean islands, accompanied by a distinct pattern of international migration. The younger population, benefiting from higher educational attainment and improved access to transportation, increasingly leaves the region to pursue professional opportunities abroad. Simultaneously, older adults are migrating to the Caribbean for retirement, contributing to an increase in the proportion of older individuals. This trend alters family dynamics and reduces the number of “available” family caregivers (1). By 2030, the number of older adults experiencing dependency in the Caribbean islands could potentially double compared to current levels (2). With the rapid growth of the older population, an increase in hospital admissions and institutionalizations is anticipated, particularly within the medico-socio-economic context of the Caribbean, where geriatric care, early detection of health issues, and broad access to healthcare continue to present significant challenges. The region’s readiness for the anticipated increase in older dependent adults is currently under scrutiny, as the Caribbean faces challenges in implementing effective strategies to address these demographic changes (3). It is crucial to understand the characteristics and timing of the demographic transition in each country, as well as the aging of populations that generally experience higher levels of social and economic inequality. These differences must be carefully considered when designing public policies. Indeed, the demographic transition will impact all Caribbean countries, though the extent will vary (4, 5). Services and housing to support dependent older adults, such as nursing homes, are currently scarce and costly in the Caribbean. Other types of care exist in the region (6), such as non-medicalized senior residences. Nevertheless, intergenerational cohabitation and foster family caregiving (7) remain the norm. Although nursing homes are not yet fully integrated into Caribbean culture, they may soon become essential for providing care to older adults. The number of nursing homes in the region has steadily increased over the past thirty years. For example, there are currently six nursing homes in Jamaica, and since the establishment of the first one in 1991, 20 have opened in Guadeloupe and 21 in Martinique (French West Indies).

Guadeloupe and Martinique are two French Caribbean territories where individuals aged 60 and over already constitute 29% of the population (8). The incidence rate of various age-related adverse outcomes, including chronic diseases (such as hypertension, diabetes, and cancer), dementia, and neuropsychiatric syndromes (4), is expected to increase dramatically. In these territories, 90% of the population is of African descent. The medical and cultural realities in these territories differ significantly from those in mainland France. The epidemiology of older adults in this region exhibits several unique characteristics that intersect with issues prevalent in both high-income European countries and middle-income Caribbean nations. Social indicators reveal that the standard of living in the French overseas territories is lower than in mainland France. In 2018, the poverty rate in Guadeloupe (French West Indies) was 34%, compared to 14% in mainland France. Additionally, the rate of severe poverty was 12% in Guadeloupe, compared to 2.1% in mainland France (9). In 2020, there were 1,301 nursing home places in Guadeloupe and 1,625 in Martinique, compared to 721 and 1,221, respectively, in 2014. Despite this increase, the number of available places remains insufficient. For instance, Guadeloupe had only 42 accommodation places per 1,000 individuals aged 75 and over, compared to 124 places in mainland France. In other high-income countries, the number of nursing home beds per 1,000 persons aged 65 and older ranges from 72.4 in Sweden to 174.5 in the United States (10). Relocating to a nursing home can offer significant benefits to residents and their families, including access to round-the-clock medical care and a secure living environment. However, admission to a nursing home is also associated with potential declines in physical status and mental health (11–13). However, the existing knowledge about nursing homes primarily derives from studies conducted outside the Caribbean, and the specific characteristics of residents in Caribbean nursing homes have not yet been addressed. Despite the anticipated increase in longevity, dependency, chronic diseases, and overall challenges in caring for older adults, coupled with a growing number of nursing homes, there remains a lack of data on nursing home residents in the Caribbean.

Extensive data are required to comprehend the healthcare trajectories of these residents and the context and challenges of dependency care in Caribbean islands. In this regard, this cross-sectional study aimed to describe, for the first time, the clinical specificities of older adults residing in nursing homes in the French West Indies.

## Methods

This study is a cross-sectional analysis of the KASEHPAD (KArukera Study of Aging in EHPAD) cohort, aimed at describing the clinical characteristics of older adults residing in nursing homes at baseline. Participants were recruited from 6 of the 41 nursing homes in Guadeloupe and Martinique. Inclusion criteria required that residents be 60 years of age or older, reside in a nursing home in Guadeloupe or Martinique, and be affiliated with French social security. The exclusion criterion was the refusal of the resident or their legal guardian to participate in the study. At baseline, healthcare professionals, including experiment geriatricians and clinical research nurses conducted interviews with the participants and their professional caregivers. The interview included physical, cognitive and psychological assessments of the participants. Professional caregivers provided information on various aspects of the participants, including sociodemographic data, medical history, medication, nutritional status, level of activity, and degree of dependence. The KASEHPAD study was registered under RGB-ID: 2020-A00960-39. Version 1.1 of the study protocol was approved by the EST 1 French Ethics Committee on June 2, 2020 (approval date: 11/05/2020). The study was also registered on ClinicalTrials.gov on October 13, 2020 (NCT04587466). Two amendments were made to the protocol: one to extend the duration of participant inclusion due to the COVID pandemic, and another to include additional centers in Martinique, as initial inclusion was planned solely for Guadeloupe. The KASEHPAD study is an observational research project involving human participants, with no identified risk to participant safety. In accordance with this classification, the requirement for a signed consent form had been waived by the regulatory authorities (Law 2012-300). Participants were provided with an information leaflet that detailed the key aspects of the study. Participation was entirely voluntary, and individuals could decline participation or withdraw at any time without any adverse consequences. Given the high prevalence of cognitive impairment among residents in nursing homes, the on-site investigator ensured that participants fully understood the implications of their involvement. In cases where a participant was under legal guardianship and/or was unable to understand the study, non-opposition from the legal guardian or a designated contact person was obtained.

### Data

This cross-sectional analysis reports the following clinical characteristics of older adults: age, gender, length of stay in nursing homes, previous residences, marital status, comorbidities and number of children.

Additionally the results of the following geriatrics scales assessed by healthcare professionals during the face-to-face interview were described:

- Nutritional status: This was evaluated using the short form of the Mini Nutritional Assessment (MNA-SF) (14). The MNA-SF consists of six questions covering anthropometric measurements (body mass index, weight loss), global assessment (mobility), and dietary questionnaire along with subjective assessment (food intake, neuropsychological problems, acute disease). A score below eight indicates undernutrition.
- Level of independence in activities of daily living (ADL): This was assessed using Katz’s scale (15), a 6-item scale that evaluates independence in six basic activities of daily living: bathing, toileting, eating, locomotion, dressing, and incontinence. For each activity, a score of 1 indicates complete autonomy, a score of 0.5 indicates partial independence, and a score of 0 indicates total dependency.
- Severity of cognitive deficits: This was assessed using the Mini-Mental State Examination (MMSE) (16). This 30-item scale evaluates the severity of cognitive impairment through items that assess orientation, learning, attention, arithmetic, memory, language, and constructive praxis. The scale is scored from 0 to 30, with lower scores indicating greater cognitive impairment.
- Physical performance: This was evaluated using the Short Physical Performance Battery (SPPB) (17). The SPPB includes three subtests that measure lower-extremity function: maintaining balance (feet side-by-side, semi-tandem, and tandem positions, each held for 10 s); walking 4 meters at a usual pace to determine gait speed (meters per second); and standing up and sitting down five times as quickly as possible with arms folded. Each subtest is scored from 0 to 4 points, with a total score ranging from 0 to 12. Higher scores reflect better physical performance. A cut-off score of ≤8 points indicates poor physical performance (18).
- Percentage of older adults confined to bed or a wheelchair: This was assessed using the Braden Scale (19). Specifically, item three of the scale evaluates the level of mobility of the participant, categorizing them as confined to bed, confined to a wheelchair, occasionally walking, or walking independently.

### Statistical analysis

Quantitative variables were expressed as mean ± standard deviation, median and range (minimum-maximum). Qualitative variables were presented as percentages. Missing values were not imputed. All analyses were performed with R v.3.0.2 software.

## Results

In the six nursing homes, 332 residents aged 60 years or older were included between September 2020 and November 2022. One participant aged 59 was excluded and 22 declined to participate, resulting in a participant rate of 93.8%. The mean age of the participants was 81.3 ± 10.1 years, with 50.5% being male. The mean weight was 62.6 ± 15.2 kg and the mean height was 1.65 ± 0.1 meter. The mean body mass index (BMI) was 23.0 ± 5.0 kg/m^2^. At the time of inclusion, participants were residing either at home (51.3%) or in a hospital or clinic (36.0%). Additionally, eight residents had come from a foster family for older adults. They had been living in the institution for an average of 4.4 ± 4.1 years. Only 10.0% of the participants were in a relationship (Table 1) and the majority of residents had no children (44.4%) or only one child (18.1%).

**Table 1:** Sociodemographic characteristics of older adults living in nursing homes in the French Caribbean nursing homes.

Characteristics	Total
Age (years) (*n* = 332)	
Mean	81.3 ± 10.1
Median (Min, Max)	81 (60–105)
% aged 75 years and more	237 (71.4%)
Mean age men	78.4 ± 9.6
Mean age women	84.2 ± 9.7
Gender (net % of men) (*n* = 332)	168 (50.6%)
Mean BMI (*n* = 287)	23.0 ± 5.0
Number of children (*n* = 315)	
No children	140 (44.4%)
One child	57 (18.1%)
≥2 children	118 (37.5%)
Length of stay in nursing homes (*n* = 307) (years)	
Mean	4.4 ± 4.1
Median (Min, Max)	3.3 (0.0–29.5)
Previous residence (*n* = 267)	
Own home	137 (51.3%)
Hospital or clinic	96 (36.0%)
Other	26 (9.7%)
Marital status (*n* = 329)	
Widowed	65 (19.8%)
In a relationship	33 (10.0%)
Single or divorced	231 (70.2%)

Regarding comorbidities, 28.3% had diabetes and 66.6% had hypertension. Heart diseases was present in 18.4% of residents, stroke in 16.3% and hemiplegia in 9.6%. Dementia was reported in 52.3% of residents and Parkinson’s disease in 9.0%. Only 14 cases of cancer were reported. The prevalence of depression was 20.2%. Of the 97 residents with visual impairment, only 26 had access to eyeglasses (Table 2).

**Table 2:** Comorbidities of older adults living in nursing homes in the French Caribbean nursing homes.

Comorbidities (*n* = 332)	*N* and %
Hypertension	221 (66.6%)
Dementia	173 (52.3%)
Visual impairment	97 (29.2%)
Diabetes	94 (28.3%)
Depression	67 (20.2%)
Heart diseases (myocardial infarction, congestive heart failure, angina)	61 (18.4%)
Stroke	54 (16.3%)
Hearing impairment	47 (14.2%)
Arthritis	44 (13.3%)
Hemiplegia	32 (9.6%)
Parkinson disease	30 (9.0%)
Tobacco use	22 (6.6%)
Cancer history	14 (4.2%)
Alcohol use	3 (0.9%)

### Geriatric assessment

The mean ADL score was 2.4 ± 2.1. The most common pattern of dependence included full assistance required for bathing (64.1%), dressing (63.8%), incontinence (54.0%) and inability to use the toilet (48.2%). Only 37.4% were able to eat without assistance, while 33.7% were completely feeding dependent. Overall, 35.8% of the participants were confined to bed or a wheelchair. The mean MMSE score was 11.3 ± 9.4 with 74.9% scoring below 18, indicating major cognitive impairment. The MNA score averaged 9.6 ± 3.6, with a quarter of the participants suffering from malnutrition (MNA score ≤ 7). Lastly, 90.0% of the participants exhibited poor physical performance, as indicated by a SPPB score of less than 8 (Table 3).

**Table 3:** Geriatrics scale scores of older adults living in nursing homes in the French Caribbean nursing homes.

Measure	Mean ± CI
MMSE score (*n* = 295)	11.27 ± 9.40
MMSE score ≤ 18	221 (74.9%)
SPPB score (*n* = 309)	2.32 ± 3.32
% of participants with SPPB score < 8	278 (90.0%)
ADL score (*n* = 326)	2.40 ± 2.11
% of participants with ADL score = 0	60 (18.4%)
Needing full assistance for bathing	209 (64.1%)
Needing full assistance for dressing	208 (63.8%)
Needing full assistance for toileting	157 (48.2%)
Needing full assistance for transferring	74 (22.7%)
Incontinence	176 (54.0%)
Total feeding dependence	110 (33.7%)
MNA score (*n* = 319)	9.56 ± 3.57
MNA score ≤ 7	88 (27.6%)
Confined to their bed or to a wheelchair (Mobility Braden scale) (*n* = 321)	115 (35.8%)

## Discussion

Baseline characteristics of the 332 residents in the KASEHPAD study provide new and general data on nursing home residents in a Caribbean region. We observed that these residents were younger compared to those in mainland France. The mean age was 81.3 years, compared to 86.1 years for residents in mainland France (20). The shortage of family caregivers may partly explain this difference. Indeed, 44% of the older adults had no children, whereas in France, this proportion is approximately 25% (21). Family solidarity is a hallmark of the Caribbean culture, which contrasts with the situation in mainland France (22). The absence of children, coupled with an international migration pattern of the young population, may contribute to the social isolation of older adults. Moreover, the scarcity of alternatives for older dependent adults like homecare solutions (i.e., mobile geriatrics teams, professional workers, telecare services) or other accommodation model like senior housing (23) could also account for this result. Life expectancy at birth is comparable between the French West Indies and mainland France. In 2020, life expectancy was 77 years for men and 83.6 years for women in Guadeloupe, compared to 79.1 and 85.1 in mainland France, respectively. Since nursing homes are relatively recent (most of them less than 20 years old) and are often converted from other types of buildings, they may not have been specifically designed to accommodate the needs of the oldest adults. In high-income countries, there is a trend of increasing mean age among nursing homes residents each year. Given the rapid demographic transition in the French West Indies, it is anticipated that the average age of residents will rise in the coming years. Finally, the relatively young age of the residents also contributes to their extended length of stay in nursing homes, which averages 4.4 years on average. Half of the residents were male, whereas generally, the usual proportion of males in nursing homes is approximately 25%. This discrepancy may be attributed to socio-anthropological factors, including the high prevalence of single parenthood and the lack of children in these territories. In the French West Indies, 40% of families are single-parent households, compared to 15% in mainland France (22).

Understanding potential differences in disease rates is essential for targeting effective interventions aimed at reducing health disparities. Dementia was diagnosed in 52.3% of the residents. This prevalence is similar to that observed in nursing homes in France and other countries (24). Nevertheless, the mean MMSE score was notably low, with three-quarters of residents, scoring 18 or below. This suggests a probable under diagnosis and under treatment of dementia. Given that residents are younger in the French West Indies, cohort studies are needed to determine whether this phenomenon is related to accelerated cognitive aging in the local population or if it is attributable to other causes, such as Parkinson’s disease or diabetes Regarding the high prevalence of Parkinson’s disease (9.1%), several studies suggests the existence of distinctive atypical parkinsonism, termed Caribbean Parkisonism, in the French Caribbean islands. This condition may involve genetic susceptibility and environmental exposures, such as the chlordecone pesticide (25–27). This atypical parkinsonism is characterized by early-onset dementia and dysautonomia. Additionally, we observed a high prevalence of cardiovascular comorbidities, including diabetes or a history of stroke. This very high prevalence of diabetes 28.4% compared to 10–20% in other French nursing homes (28, 29) is a notable characteristic of the Caribbean population (30) and the French West Indies. Indeed, the prevalence of diabetes in the general population is estimated at 10.2% in Guadeloupe (31). Studying the management of diabetes and the pharmacological treatments used in nursing homes could therefore contribute to improving practices. Conversely, our study observed a low prevalence of active cancers or a history of cancer (4.2%) dy. The low prevalence of cancer is characteristic of these territories, with the exception of prostate cancer (32). In international nursing homes, this prevalence is typically twice as high (33, 34). Cancer is likely underdiagnosed in Caribbean nursing homes although the effectiveness of cancer screening in these settings remains a subject of debate (35).

Severe dependency was also exceptionally high, with 35.8% of residents confined to bed or a wheelchair. In comparison to studies conducted in French nursing homes, the mean ADL score in our study was 2.4 ± 2.1, whereas it was 2.8 ± 2,05 (28) and 3.3 ± 1.6 (29) in other studies. The sub-scores for each activity (toileting, dressing, incontinence, transferring and eating) were 10% higher than those reported in other studies. Finally, we observed that a quarter of residents were malnourished according to the MNA-SF scale (score ≤ 7). This rate is higher than the means reported in other studies of residents in long-term care facilities (36). This difference warrants further attention, as the management of nutrition is one of the most frequent challenges in nursing homes and has been associated with an increased risk of adverse health outcomes (37).

The rising demand for long-term care services is occurring in a context of limited supply in the Caribbean region. It is crucial to understand and analyze the experiences of territories that are further along in the demographic transition. The strength of this work lies in its focus on a unique region (the Caribbean, with a population of approximately 44 million inhabitants). The French West Indies are officially part of a high-income country (France) ensuring specific standards, yet they remain, from a sociocultural and environmental perspective, aligned with low and middle-income countries. In this context, this work offers a unique opportunity to provide data bridging the two worlds and could serve as a living lab/testing field for translating research evidence (frequently coming from high-resourced settings) into practice, where resources and dynamics may differ.

One of the main limitations of this study is the lack of access to data from informal caregivers, who could provide a different insight on the participants’ psychosocial condition. Another potential limitation relates to the study’s follow-up study period. The inclusion and follow-up of most residents occurred during COVID-19 pandemic, which may have influenced numerous variables, including mortality, hospitalizations and all quality of life related factors. For this reason, we have amended the study protocol to include additional older adults in our cohort in Martinique since April 2022, following the peak of COVID infections. Due to the absence of nursing homes in most Caribbean countries and the lack of data on the profiles of dependent older adults in the region, the results of our study may not be fully extrapolatable to other Caribbean territories. Finally, it may be necessary to conduct sociological studies to gain a deeper understanding of the place of older adults and the acceptance of the nursing homes model within the Caribbean culture.

Studies are necessary to enhance the care of older adults living in Caribbean nursing homes. The KASEHPAD study provides valuable insights into the clinical profile of older adults. Residents in Caribbean nursing homes appear to be younger to those in metropolitan areas, particularly concerning dementia or disabilities. The 1-year data from the KASEHPAD study will be instrumental in evaluating the Caribbean model of nursing homes concerning health events such as mortality, hospitalization, and quality of life. This assessment will guide the adaptation of training for local stakeholders and the improvement of care provision and its organization at the local level. This work could assist other Caribbean countries in designing future long-term care systems, whether through the implementation of nursing homes or other models of care for dependent older adults. The potential benefits of this study include supporting evidence-informed public health policies and introducing greater rationality into social and economic debates, which are often driven by personal experiences or extrapolated indicators from other countries, rather than reflecting the realities of the Caribbean context.

## Data Availability

The raw data supporting the conclusions of this article will be made available by the authors, without undue reservation.
